# Additively Manufactured Zn‐2Mg Alloy Porous Scaffolds with Customizable Biodegradable Performance and Enhanced Osteogenic Ability

**DOI:** 10.1002/advs.202307329

**Published:** 2023-12-07

**Authors:** Xuan Wang, Aobo Liu, Zhenbao Zhang, Dazhong Hao, Yijie Liang, Jiabao Dai, Xiang Jin, Huanze Deng, Yantao Zhao, Peng Wen, Yanfeng Li

**Affiliations:** ^1^ Postgraduate Training Base Jinzhou Medical University and The Fourth Medical Centre Chinese PLA General Hospital Beijing 100048 China; ^2^ Department of Stomatology the Fourth Medical Centre Chinese PLA General Hospital Beijing 100048 China; ^3^ State Key Laboratory of Tribology in Advanced Equipment Beijing 100084 China; ^4^ Department of Mechanical Engineering Tsinghua University Beijing 100084 China; ^5^ Senior Department of Orthopedics the Fourth Medical Centre PLA General Hospital Beijing 100048 China; ^6^ Beijing Engineering Research Center of Orthopedics Implants Beijing 100048 China

**Keywords:** additive manufacturing, biodegradable performance, osteogenic ability, structure design, Zn‐Mg alloy

## Abstract

The combination of bioactive Zn‐2Mg alloy and additively manufactured porous scaffold is expected to achieve customizable biodegradable performance and enhanced bone regeneration. Herein, Zn‐2Mg alloy scaffolds with different porosities, including 40% (G‐40‐2), 60% (G‐60‐2), and 80% (G‐80‐2), and different unit sizes, including 1.5 mm (G‐60‐1.5), 2 mm (G‐60‐2), and 2.5 mm (G‐60‐2.5), are manufactured by a triply periodic minimal surface design and a reliable laser powder bed fusion process. With the same unit size, compressive strength (CS) and elastic modulus (EM) of scaffolds substantially decrease with increasing porosities. With the same porosity, CS and EM just slightly decrease with increasing unit sizes. The weight loss after degradation increases with increasing porosities and decreasing unit sizes. In vivo tests indicate that Zn‐2Mg alloy scaffolds exhibit satisfactory biocompatibility and osteogenic ability. The osteogenic ability of scaffolds is mainly determined by their physical and chemical characteristics. Scaffolds with lower porosities and smaller unit sizes show better osteogenesis due to their suitable pore size and larger surface area. The results indicate that the biodegradable performance of scaffolds can be accurately regulated on a large scale by structure design and the additively manufactured Zn‐2Mg alloy scaffolds have improved osteogenic ability for treating bone defects.

## Graphical abstract

Ideal orthopedic implants should have the customizable biodegradable performance and good osteogenic ability for the specific patient. Here, structure design has been successfully utilized to customize the biodegradable performance and enhance the osteogenic ability of additively manufactured Zn‐2Mg scaffolds. Additionally, the mechanism of how structure design influences the performance of biodegradable Zn‐2Mg scaffolds is thoroughly clarified.

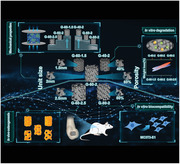

## 1 Introduction

Biomaterial implant therapy is effective for repairing bone defects caused by tumors or external trauma.^[^ ^1^ ^]^ A wide variety of biomaterials such as ceramics, polymers, glass, and metals have been extensively studied as potential orthopedic implant materials.^[^ ^2^ ^]^ Among these, biometals are frequently utilized in load‐bearing situations, due to their sufficient mechanical strength, ductility, and fatigue resistance.^[^ ^3^, ^4^, ^5^, ^6^, ^7^ ^]^ Commonly used biometals like stainless steel, cobalt, and titanium (Ti) alloys are bio‐inert and remain in the body for a life‐long time. The surgery for the removal of bio‐inert implants imposes a considerable physical and psychological burden on patients. Moreover, these materials can release harmful ions, metal salts, or wear particles during corrosion or wear, resulting in allergic and inflammatory reactions.^[^ ^8^, ^9^, ^10^ ^]^ Biodegradable metals (BMs) introduce a promising solution to this dilemma due to their good biodegradability and biocompatibility.^[^ ^11^ ^]^ BMs are mainly composed of three families, magnesium (Mg)‐based BMs, iron (Fe)‐based BMs, and zinc (Zn)‐based BMs.^[^ ^12^, ^13^ ^]^

Among all BMs, Zn has a moderate corrosion rate. And there is no release of gas during its degradation. Moreover, Zn‐based BMs exhibit good antibacterial properties and osteogenic potential, leading to their recognition as promising materials for bone implants.^[^ ^14^, ^15^, ^16^, ^17^, ^18^ ^]^ However, the mechanical strength of pure Zn is insufficient, and its biocompatibility requires further improvement.^[^ ^19^ ^]^ To optimize its comprehensive properties, researchers have employed the alloying method.^[^ ^20^ ^]^ Li et al. demonstrated that the addition of Mg element to pure Zn greatly enhanced the mechanical properties, corrosion behavior, and biocompatibility.^[^ ^21^ ^]^ Yang et al. investigated the mechanical properties, corrosion behavior, and cellular reactions of binary Zn alloys with alloying elements of Mg, Ca, Sr, Li, Mn, Fe, Cu, and Ag, respectively. It was demonstrated that Zn‐Mg showed the desirable combination of properties for bone implants.^[^ ^22^ ^]^ The addition of Mg refined the grains, and the precipitation phase Mg_2_Zn_11_ significantly strengthened the matrix, thereby enhancing the mechanical properties of Zn‐Mg alloys.^[^ ^14^, ^23^, ^24^ ^]^ Furthermore, the release of Mg ions effectively improved cytocompatibility and osseointegration.^[^ ^25^ ^]^ Therefore, Zn‐Mg alloys are seen as an ideal material for orthopedic implants. For orthopedic implants, in addition to proper material selection, they should also meet specific requirements in geometry and structure. They require a personalized, heterogeneous morphology that matches the bone defects. Also, these implants should have a porous structure for two reasons: first, the porous structure reduces the implant's elastic modulus, thereby mitigating the stress shielding effect. Second, it provides conditions for osteoblast and mesenchymal cell migration, proliferation, and vascularization, promoting bone growth and establishing a strong mechanical connection with the surrounding bone tissue. Nevertheless, when it comes to the fabrication of porous scaffolds, conventional manufacturing techniques like casting and forging encounter difficulties in producing such complex structures.

Additive manufacturing (AM), commonly known as “Three‐dimensional (3D) printing”, fabricates objects from 3D digital models by depositing materials layer by layer.^[^ ^26^ ^]^ It has advantages such as free structural design, less material waste, and short lead time, providing a way to fabricate components with complex geometry and structure. Among all AM techniques, laser powder bed fusion (L‐PBF) proves effective for the fabrication of orthopedic implants due to its high precision.^[^ ^27^, ^28^ ^]^ In recent years, L‐PBF technology has realized the reliable production of Zn‐Mg scaffolds. Qin et al. manufactured Zn‐Mg alloy porous scaffolds by L‐PBF technology and found that the addition of Mg successfully increased the biocompatibility of scaffolds and enhanced bone formation in rabbit femurs. However, the new bone tissue appeared around the scaffold and did not grow significantly into the interior of the scaffold.^[^ ^29^ ^]^ Zhao et al. also found that Zn‐Mg alloy porous scaffolds exhibited higher mechanical strength and faster degradation rates than pure Zn porous scaffolds. Moreover, Zn‐Mg alloy scaffolds had better osteogenic activity than pure Zn scaffolds. This suggested that alloying with Mg was an effective method to improve the comprehensive properties of Zn‐based BM scaffolds. However, the newly formed bone tissue did not significantly grow into the pores of the two scaffolds, indicating a need for the further improvement of osteogenic ability.^[^ ^30^ ^]^

Ideal orthopedic implants should have the customizable biodegradable performance and good osteogenic ability for the specific patient. The parameters of structure design, such as porosity and unit size, can significantly influence the properties of scaffolds. It is promising to obtain the required performance of scaffolds by adjusting the porous design. Li et al. designed and manufactured pure Zn scaffolds with different porosities. The weight loss of scaffolds was 7–12% after in vitro immersion, depending on the structure design. The mechanical strength of scaffolds decreased with the increasing porosity.^[^ ^31^ ^]^ Liu et al. designed and fabricated Zn‐0.8Li‐0.1Mg porous scaffolds with porosities of 60%, 70%, and 80%, respectively, and investigated the effects of porosity on their mechanical properties, in vitro degradation properties, biocompatibility, and osteogenic properties. The results showed that the mechanical strength of scaffolds decreased with the increasing porosities. The higher porosity indicated the higher weight loss due to the increased specific surface area and permeability. In vitro biocompatibility and osteogenic ability were also influenced by structure design, which affected the amount of released ions.^[^ ^32^ ^]^ The in vitro studies above show the possibility of modifying the properties of Zn‐based BM scaffolds by structure design. However, it's important to acknowledge that the results of in vitro tests may not accurately predict in vivo performance due to the substantial differences between the in vitro test condition and the actual in vivo environment. While, until now, no study has been conducted on the impact of structure design on the in vivo performance of biodegradable Zn‐based BM scaffolds. The mechanism that how structure design controls the biodegradable performance and osteogenic ability of Zn‐based BM scaffolds remains unclear. Therefore, in this study, Zn‐2Mg alloy scaffolds with different porosities, including 40%, 60%, and 80%, and different unit sizes (1.5, 2, and 2.5 mm) were designed fabricated to systematically study the influence and the underlying influencing mechanism of structure design on in vivo behavior of Zn‐based BM scaffolds for the first time. The mechanical properties, in vitro biodegradation behavior, biocompatibility, and osteogenic ability of scaffolds were also reported. This study offers solid evidence for adjusting the structure design of biodegradable metal scaffolds to obtain customizable biodegradable performance and enhanced bone regeneration.

## 2 Results

### 2.1 Mechanical Properties

**Figure 1:**
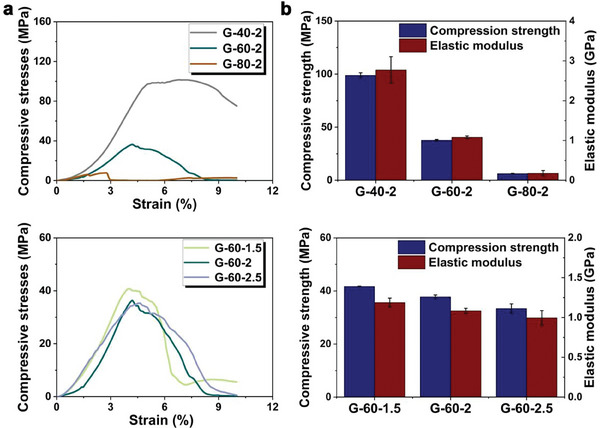
Mechanical properties of Zn‐2Mg scaffolds with different structure designs: a) compressive curves and b) analysis of compressive strength and elastic modulus (n = 3).

**Figure** **1** **a** showed the compressive stress‐strain curves of Zn‐2Mg scaffolds. All curves showed obvious fluctuation, indicating the poor ductility of Zn‐2Mg alloys. As shown in Figure 1b, the compressive strength (CS) and elastic modulus (EM) of G‐40‐2, G‐60‐2, and G‐80‐2 were 98.86 ± 2.39, 37.77 ± 0.70, 6.29 ± 0.37 MPa, and 2.77 ± 0.33, 1.08 ± 0.03, 0.18 ± 0.06 GPa, respectively. Notably, porosity significantly affected the mechanical properties. CS and EM decreased with the increasing porosities. As for scaffolds with different unit sizes, the CS and EM of G‐60‐1.5, G‐60‐2, and G‐60‐2.5 were 41.70 ± 0.13, 37.77 ± 0.70, 33.38 ± 1.79 MPa and 1.19 ± 0.06, 1.08 ± 0.03, and 0.99 ± 0.09 GPa, respectively. CS and EM decreased with increasing unit sizes.

### 2.2 In Vitro Degradation Behavior

**Figure 2:**
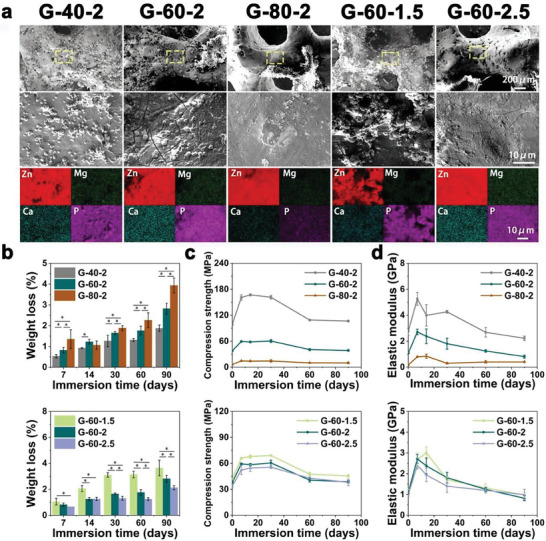
In vitro degradation behavior of Zn‐2Mg scaffolds in Hank's solution: a) SEM images and EDS elemental analysis of the surface of the scaffold after 90 days of immersion, b) weight loss analysis of scaffolds (n = 3, *p < 0.05), c,d) compressive strength and elastic modulus after degradation (n = 3).

As shown in Figure S1 (Supporting Information), after the immersion test of 90 days, degradation products were observed on the surface of the scaffolds. The structural integrity of all scaffolds remained intact without obvious damage. The degradation products had many morphologies, such as sphericity, clusters, and granules, as shown in **Figure** **2**a. Degradation products were mainly composed of Zn, Mg, Ca, and P, as detected by EDS. Figure 2b showed the weight loss of all scaffolds. The weight loss increased with the increasing immersion time. After the immersion of 90 days, the weight loss of G‐40‐2, G‐60‐2, G‐80‐2, G‐60‐1.5, and G‐60‐2.5 were 1.87 ± 0.15%, 2.82 ± 0.26%, 3.93 ± 0.35%, 3.64 ± 0.61%, and 2.13 ± 0.16%. For scaffolds with different porosities, the weight loss ranked as G‐80‐2 > G‐60‐2 > G‐40‐2. As for scaffolds with different unit sizes, the weight loss decreased with the increasing unit sizes. The pH of scaffolds fluctuated between 6.86 and 7.91 during the degradation process (Figure S2, Supporting Information). Compressive behaviors of scaffolds after immersion tests were evaluated as shown in Figure 2c and d. The CS of all scaffolds increased significantly after being immersed in Hank's for 7 days. CS of G‐40‐2, G‐60‐2, G‐80‐2, G‐60‐1.5, and G‐60‐2.5 were enhanced to 160.94 ± 6.19, 59.34 ± 0.99, and 14.39 ± 0.68, 65.95 ± 0.93, and 52.25 ± 5.29 MPa, respectively. And then, CS showed no significant change from day 7 to 30. After day 30, CS was significantly decreased. Interestingly, after 90 days of the degradation experiment, the strength of the scaffolds was comparable to the as‐built scaffolds. As shown in Figure 2d, for EM of all scaffolds, an increase in the initial stage during immersion and a subsequent decrease of EM were observed, which was similar to the change of CS.

### 2.3 In Vitro Biocompatibility

**Figure 3:**
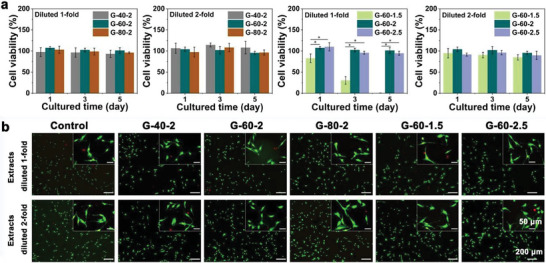
In vitro cytocompatibility of Zn‐2Mg scaffolds: a) cytotoxicity testing of MC3T3‐E1 cells after 1, 3, 5 days of incubation with extracts (n = 3, *p < 0.05), b) live/dead staining of MC3T3‐E1 cells after 24 hours of incubation with extracts.

CCK‐8 method was utilized for assessing the cell viability of MC3T3‐E1 cells, co‐cultured with sample extracts (**Figure** **3**a). Scaffolds with different porosities had cell viability around 100% at both 1‐fold and 2‐fold diluted extracts, indicating good cytocompatibility. As for scaffolds with different unit sizes, at 1‐fold diluted extracts, the cell viability of G‐60‐1.5 reached 82.70 ± 10.28% on the first day. However, the cell viability decreased with increasing incubation time, and on day 5, almost all cells died, showing significant cytotoxicity. While the rest of the groups showed acceptable cytocompatibility. When the extracts were further diluted to 2‐fold, all groups showed good cytocompatibility, with cell viability ranging from 85.01 ± 6.41% to 103.99 ± 5.26%. The live‐dead staining results were shown in Figure 3b. Co‐cultured with the extracts for 24 h, G‐60‐1.5 at 1‐fold diluted extracts showed fewer live cells compared with other groups. When using 2‐fold extracts, all groups had good cell morphology and a comparable amount of live cells, which was consistent with CCK‐8 results.

**Figure 4:**
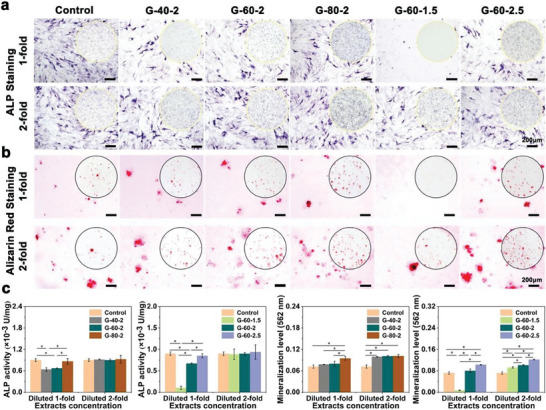
In vitro osteogenic differentiation of Zn‐2Mg scaffolds: a) ALP staining on day 14, b) Alizarin Red staining on day 21, c) quantitative and semi‐quantitative analysis of ALP activity and mineralization levels (n = 3, *p < 0.05).

The osteogenic differentiation and mineralization of MC3T3‐E1 cells were detected by ALP and alizarin red staining. As shown in **Figure** **4**a and b, cells in the G‐60‐1.5 group were almost dead in the 1‐fold diluted extract. The osteogenic ability of G‐80‐2 and G‐60‐2.5 was the best in scaffolds with different porosities and unit sizes respectively. For the 2‐fold diluted extracts, the osteogenic differentiation levels did not differ significantly between the groups. As for mineralization level, in scaffolds with different unit sizes, it ranked as G‐60‐2.5 > G‐60‐2 > G‐60‐1.5. While no significant difference in mineralization levels was observed in scaffolds with different porosities. The same trend was obtained by quantitative and semi‐quantitative calculations of ALP activity and mineralization levels, as shown in Figure 4c.

### 2.4 In Vivo Animal Experiments

#### 2.4.1 Micro‐CT Analysis

**Figure 5:**
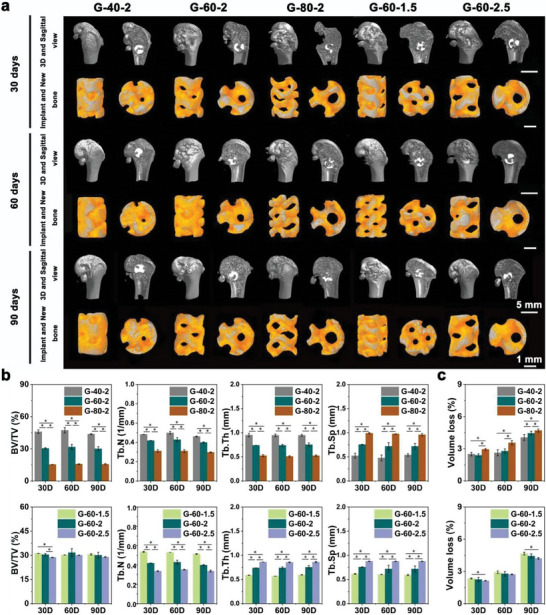
Evaluation of osteogenic ability of Zn‐2Mg scaffolds: a) Micro‐CT images of implantation including new bone and implants, b) quantitative analysis of osteogenic ability (BV/TV, Tb. N, Tb. Th, and Tb. Sp) (n = 3, *p < 0.05), and c) volume loss analysis of Zn‐2Mg scaffolds (n = 3, *p < 0.05).

As shown in **Figure** **5**a, new bone tissue not only formed around the scaffolds but also grew into their pores. And the amount of new bone increased with time. Quantitative analysis of the four osteogenic indices obtained from Micro‐CT was shown in Figure 5b. Compared with the G‐60‐2, and G‐80‐2 groups, higher BV/TV, Tb. N, Tb. Th, and lower Tb. Sp were found in the G‐40‐2 group, indicating the better in vivo osteogenic ability of the G‐40‐2 group. For the groups with different unit sizes, the BV/TV showed no significant difference. However, compared with G‐60‐2 and G‐60‐2.5, higher Tb. N and lower Tb. Sp, Tb. Th were found in the G‐60‐1.5 group, suggesting thinner, more numerous, and denser newborn bone trabeculae formed in G‐60‐1.5.

The volume loss of scaffolds in vivo was shown in Figure 5c. For the groups with different porosities, the G‐80‐2 exhibited the highest volume loss, which was 4.70 ± 0.14% on day 90 after implantation, approximately 1.16 times that of the G‐40‐2. In the groups with different unit sizes, the highest volume loss was observed in the G‐60‐1.5 group. On day 90, the volume loss of G‐60‐1.5 was 4.66 ± 0.13%, which was 0.44% higher than that of G‐60‐2.5. The results were similar to in vitro degradation tests and indicated that the higher porosity and smaller unit size resulted in higher volume loss of scaffolds.

#### 2.4.2 Histological Evaluation

**Figure 6:**
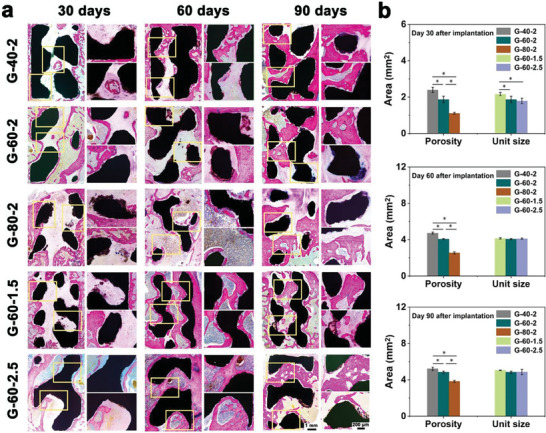
Zn‐2Mg scaffolds implanted in vivo for 30, 60, and 90 days: a) full‐view and magnified images of hard‐tissue, b) new bone tissue area analysis (n = 3, *p < 0.05).

Hard tissue sections were shown in **Figure** **6**a. According to the full‐view images, the amount of new bone increased over time in all groups, on day 90 after implantation, new bone tissue not only emerged around the scaffold but also exhibited significant growth into the scaffold. Better bone growth was observed in the G‐40‐2 group in scaffolds with different porosities, while no significant differences were observed in scaffolds with different unit sizes. In the magnified images, compared with the G‐80‐2 group, better osseointegration was observed in the G‐40‐2 and G‐60‐2 groups on day 90 after implantation. Figure 6b showed the new bone tissue area in scaffolds with varying structure designs. Among scaffolds with different porosities, G‐40‐2 exhibited the largest area of new bone tissue. Specifically, on day 90 after implantation, the new bone tissue areas for G‐40‐2, G‐60‐2, and G‐80‐2 were measured at 5.22 ± 0.16, 4.84 ± 0.11, and 3.83 ± 0.11 mm^2^, respectively. Regarding scaffolds with different unit sizes, the new bone tissue area in the G‐60‐1.5 was 2.17 ± 0.08 mm^2^ on day 30 after implantation, while G‐60‐2.5 demonstrated a smaller area of 1.79 ± 0.15 mm^2^. However, no significant differences were observed in the new bone tissue areas among scaffolds with different unit sizes on day 60 and 90 after implantation. These findings were consistent with the Micro‐CT results. The in vivo biosafety of scaffolds was evaluated by histologic sections of vital organs. As shown in Figure S3 (Supporting Information), no histopathological changes were observed in the sections of the heart, liver, spleen, lung, and kidney in all groups, indicating that all scaffolds had acceptable in vivo biosafety.

## 3 Discussion

### 3.1 Mechanical Properties

Orthopedic implants should have adequate strength and appropriate elastic modulus. Inadequate strength leads to the fracture of the implant, and the elastic modulus mismatch can trigger a stress shielding effect. The strength of pure Zn is inferior considering its applications in load‐bearing occasions. Alloying can significantly enhance the mechanical strength of pure Zn.^[^ ^35^ ^]^ Our previous research found that Zn‐2Mg fabricated by L‐PBF had a pronounced tensile strength of 283 MPa,^[^ ^29^ ^]^ successfully dealing with the low‐strength issue of pure Zn. As for elastic modulus, Zn alloys usually have elastic modulus ranging from 94 to 110 GPa.^[^ ^36^ ^]^ However, the human cancellous bone has a modulus of 0.3 – 3.2 GPa, much lower than that of Zn alloys.^[^ ^37^ ^]^ A porous structure is an effective way to reduce the modulus of Zn‐based BMs.^[^ ^38^, ^39^ ^]^ Li et al. fabricated porous pure Zn scaffolds with a diamond unit. The elastic modulus of the scaffolds decreased to 0.79 GPa.^[^ ^40^ ^]^ The diamond unit has been widely used in Zn‐based BM scaffolds. However, the unit contains sharp transitions between struts, where stress concentration occurs, leading to early fracture near the joints. In recent years, unit cells based on triply periodic minimal surface (TPMS), which have zero mean curvature and large surface areas, have attracted great attention in the field of bone implants.^[^ ^41^, ^42^, ^43^ ^]^ TPMS‐based scaffolds have smooth transitions, thus leading to a uniform stress contribution. Moreover, TPMS‐based scaffolds have topological characteristics and curvature similar to those of human bone trabeculae, which can also improve tissue regeneration.^[^ ^44^, ^45^, ^46^, ^47^, ^48^ ^]^

**Figure 7:**
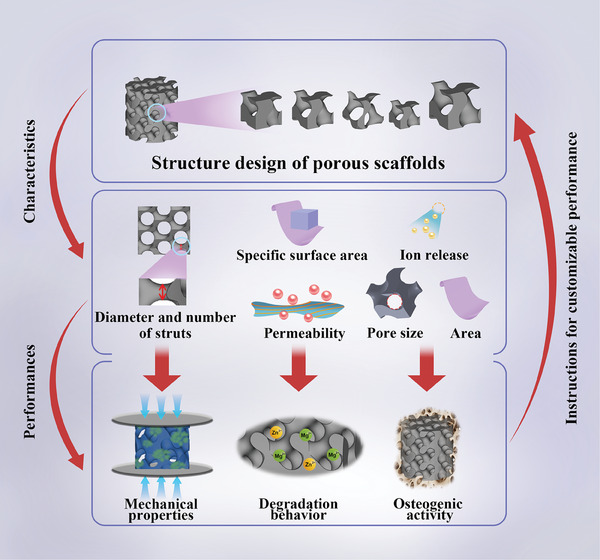
Schematic diagram of mechanisms of how structure design controls the performance of scaffolds.

Porosity and unit size are crucial geometric parameters for TPMS scaffolds, which significantly influence their mechanical properties.^[^ ^49^, ^50^, ^51^, ^52^, ^53^ ^]^ As discussed in Section 2.1, the mechanical strength of scaffolds decreased with the increase of porosities, following the well‐known Ashby‐Gibson law.^[^ ^54^ ^]^ The CS and EM of G‐40‐2 were 98.9 MPa and 2.8 GPa respectively, 15.7 and 15.4 times those of G‐80‐2. When increasing the porosity from 40% to 80%, the strut diameter of scaffolds decreased from 1.06 to 0.52 mm, thus resulting in the deterioration of the resistance to load and deformation. In terms of unit sizes, the strength of scaffolds decreased with the increasing unit sizes. The CS of G‐60‐1.5 was 41.70 ± 0.13 MPa, higher than the 33.38 ± 1.79 MPa of G‐60‐2.5. Yang et al. fabricated pure Ti scaffolds with different unit sizes. It was found that an increase in unit size from 3.5 to 5.5 mm resulted in a decrease in Young's modulus of the scaffolds from 673.08 to 518.71 MPa and a decrease in the compressive strength from 11.43 to 7.73 MPa.^[^ ^55^ ^]^ Yang et al. obtained the same result in Ti alloys. The strength increased from 31.3 MPa to 38.79 MPa when the unit size was reduced from 5 to 3 mm. These results are in line with this study. For different unit sizes, although the large unit size of scaffolds indicates the increased strut diameter, the number of units is reduced, weakening the constraints between the units, increasing the length of the strut bending region, and ultimately reducing the resistance to bending deformation as shown in **Figure** **7**.^[^ ^56^ ^]^ Therefore, the mechanical properties of scaffolds greatly depend on porosities and unit sizes. In real clinical treatments, the mechanical behavior of implants should be customized for the specific patient. The precise control of the structure design of scaffolds provides a way for obtaining customizable mechanical performance.

### 3.2 Degradation Behavior

As discussed in Section 2.2 and 2.4, the degradation behavior was greatly influenced by the porosity and unit size of scaffolds. A decrease in porosity from 80% to 40% in vitro led to a reduction in weight loss from 3.93% to 1.87%, while an increase in unit size from 1.5 to 2.5 mm resulted in decreased weight loss from 3.64% to 2.13%. Similar trends were also observed in in vivo tests. As reported by Liu et al., scaffolds with different structure designs had different biodegradable behaviors due to variations in specific surface area and permeability.^[^ ^32^ ^]^ A larger specific surface area indicated more area exposed to the corrosion medium per volume, thus increasing the weight loss. Higher permeability resulted in the elevated capability for the exchange of medium across scaffolds, leading to a higher degradation rate (Figure 7). As shown in **Table** **1**, the specific surface area increased with the increasing porosity. In terms of permeability, lots of previous studies had shown that the porosity of scaffolds was proportional to permeability.^[^ ^31^, ^57^ ^]^ The scaffolds with higher porosity had larger specific surface area and higher permeability, thus leading to higher weight loss. As for scaffolds with different unit sizes, although the scaffolds with larger unit sizes had higher permeability, the specific surface area of G‐60‐1.5, G‐60‐2, and G‐60‐2.5 was 6.08, 4.81, and 4.08 mm^−1^, decreasing with increased unit sizes, thus explaining the lower weight loss at the larger unit size. In summary, the weight loss can be effectively increased by increasing the porosity or reducing the unit size. The biodegradation behavior can be customized by changing the structure designs of the porous scaffolds.

**Table 1:** Structural characteristics of Zn‐2Mg scaffolds with five different structure designs.

Structural characteristics		G‐40‐2	G‐60‐2	G‐80‐2	G‐60‐1.5	G‐60‐2.5
Designed	Area [mm^2^]	360	327	251	414	278
	Specific surface area [mm^−1^]	3.56	4.81	7.38	6.08	4.08
	Unit size [mm]	2	2	2	1.5	2.5
	Pore size [mm]	0.58	0.83	1.12	0.63	1.04
	Strut diameter [mm]	1.06	0.80	0.52	0.60	1.00
	Porosity [%]	40.46	59.91	79.96	59.92	59.92
Measured	Porosity [%]	38.34 ± 1.89	59.33 ± 0.89	81.73 ± 0.67	56.38 ± 1.76	57.56 ± 1.92

The change in mechanical strength was also observed during degradation. As discussed in Section 2.2, an obvious increased strength and modulus were detected at the early stage of in vitro degradation, explained by the adherence of degradation products, which had a hardness five times that of the scaffolds.^[^ ^40^, ^58^, ^59^ ^]^ However, with the immersion time passing by, the mechanical strength was decreased due to the detachment of degradation products and degradation of scaffolds. It was worth noting that, after the 3‐month degradation experiment, the mechanical strength of scaffolds was comparable to that of the as‐built samples. Therefore, Zn‐2Mg porous scaffolds can provide the required mechanical support without chipping or fracturing during the 3‐month defect repair period.

### 3.3 Osteogenic Activity

As shown in Figure 5a, all scaffolds with different structure designs showed good osteogenic ability. New bone grew into the pores of the scaffolds after the implantation, indicating that the Zn‐2Mg scaffolds in this study had a significantly enhanced osteogenic ability, due to the selection of Zn‐2Mg alloy and gyroid structure. The Mg ions released from Zn‐2Mg alloys enhance cell proliferation and adhesion.^[^ ^29^ ^]^ The TPMS gyroid structure has topological and curvature characteristics similar to those of human bone trabeculae, improving tissue regeneration and providing a large surface area and permeability to promote cell adherence and retention.^[^ ^47^, ^60^ ^]^ Moreover, the osteogenic ability can be further optimized by adjusting the structure design of scaffolds. Different osteogenic abilities of scaffolds were observed in different groups, as discussed in Section 2.4. A significantly increased amount of new bone was found in the scaffolds with decreased porosities. Slightly more bone formation was found in scaffolds with decreased unit sizes. Osteogenesis in vivo can be influenced by many factors. Scaffolds with different structure designs show different osteogenic abilities due to their different physical, and chemical characteristics.

For physical characteristics, pore size and surface area of scaffolds change with varying porosities and unit sizes. The pore size has a significant impact on the biocompatibility and osteogenic activity of scaffolds.^[^ ^61^ ^]^ Pore sizes of 400 – 600 µm are regarded as favorable for osteogenesis and osseointegration.^[^ ^62^ ^]^ Taniguchi et al. studied the influence of pore size on the performance of L‐PBF Ti scaffolds. The results showed that scaffolds with a pore diameter of 600 µm showed superior osteogenic activity than the scaffolds with pore sizes of 300 and 900 µm.^[^ ^63^ ^]^ Fukuda et al. implanted Ti scaffolds with pore sizes of 500, 600, 900, and 1200 µm into beagle dogs, and the results showed that the osteoinductive effect was stronger with pore sizes of 500 and 600 µm.^[^ ^64^ ^]^ It deserves to be noted that either too large or too small pore size has some negative effects on cell proliferation, differentiation, and bone regeneration.^[^ ^61^ ^]^ Theoretically, the larger the pore size of the scaffold is, the easier the transport of oxygen and nutrients is, thus promoting cell proliferation, differentiation, and intercellular signaling as well as blood vessel formation. However, the velocity of cells passing through the center of the pore increases significantly with increasing pore sizes, which leads to a significant increase in fluid velocity and vortex formation in the center of the scaffold. This phenomenon leads to energy dissipation, which may affect the cell inoculation of the scaffold. While, a small pore size impedes the transportation of blood and nutrients, resulting in poor osteoblast growth. The pore sizes of G‐40‐2 and G‐60‐1.5 were 580 and 630 µm, approximately in the range of 400 – 600 µm, thus explaining the better osteogenesis. When increasing porosities and unit sizes, the pore sizes increased beyond 600 µm, leading to weakened osteogenesis.

In addition to pore size, the surface area of scaffolds plays an important role in determining osteogenesis.^[^ ^65^ ^]^ Cell adhesion after implantation is a crucial process for bone healing due to its influence on subsequent cell proliferation, differentiation, and tissue formation.^[^ ^66^ ^]^ Increased surface area provides more locations for cell‐surface interactions, contributing to higher osteoinductive protein adsorption as well as ion exchange, providing more space for cell adhesion and proliferation.^[^ ^67^ ^]^ The largest surface area of G‐40‐2 and G‐60‐1.5 in scaffolds with different porosities and unit sizes respectively also explained their enhanced osteogenic ability.

For biodegradable scaffolds, different from bio‐inert ones, the types and amounts of released ions during degradation, named as chemical characteristics, also influence the osteogenic ability. Zn and Mg ions are released during the degradation of Zn‐2Mg. Zn^2+^ has a dose‐dependent effect on the biocompatibility of MC3T3‐E1 cells. High concentrations of Zn^2+^ lead to low cell viability and bad cell morphology. While low concentrations of Zn^2+^ promote cell proliferation. Mg^2+^ can effectively enhance biocompatibility and osteogenesis. The ion concentrations of Mg^2+^ and Zn^2+^ in extracts were measured as shown in Figure S4 (Supporting Information). There was no significant difference in Mg^2+^ concentration among all five groups. The amount of released Zn^2+^ differed in different scaffolds due to the different surface areas and permeabilities. G‐60‐1.5 had the highest concentration of released Zn^2+^, resulting in significant cytotoxicity, which explained the bad cytocompatibility and osteogenic capacity at the 1‐fold extracts in vitro. When the extracts were further diluted to 2‐fold, the concentration of Zn^2+^ greatly decreased, and the negative effect of Zn^2+^ was weakened. Therefore, all groups shared similarly good biocompatibility and osteogenic activity.

Different from in vitro tests, the liquid circulation in vivo effectively reduced the concentration of ions around the implant.^[^ ^68^, ^69^, ^70^ ^]^ The concentration of ions in vivo could be much lower than that of the in vitro extract. Therefore, like the in vitro results at 2‐fold diluted extracts, due to the limited concentration of released ions, the effect of chemical characteristics on the in vivo performance of Zn‐2Mg scaffolds was not significant compared to physical characteristics. In summary, structure design can significantly influence the osteogenic ability of Zn‐based BM scaffolds. It is promising that the osteogenic ability of scaffolds can be optimized by adjusting the structure design (Figure 7).

## 4 Conclusion 

In this study, Zn‐2Mg alloy scaffolds with different porosities and unit sizes were successfully fabricated using L‐PBF technology. The effects of porosity and unit size on the mechanical properties, in vitro and in vivo degradation behavior, cytocompatibility, and osteogenic activity of Zn‐2Mg alloy scaffolds were systematically investigated. The main conclusions were shown as follows.

1. The mechanical properties of scaffolds could be effectively controlled by changing porosities and unit sizes. With the same unit size, the compressive strength and elastic modulus of scaffolds substantially decreased with increasing porosities. The CS and EM of G‐40‐2 were 98.9 MPa and 2.8 GPa, 15.7 and 15.4 times those of G‐80‐2. With the same porosity by contrast, the CS and EM just slightly decreased with increasing the unit sizes.
2. Degradation behaviors of scaffolds were significantly influenced by the porosity and unit size, which was majorly explained by their various specific surface areas and permeabilities. Among scaffolds with different porosities, the weight loss increased with the increased porosities. The weight loss of G‐80‐2 was ≈2.1 times that of G‐40‐2. For scaffolds with different unit sizes, the weight loss increased with the decreased unit sizes. The weight loss of G‐60‐1.5 was about 1.7 times that of G‐60‐2.5.
3. The additively manufactured Zn‐2Mg scaffolds showed improved osteogenic ability for treating bone defects. The osteogenic ability of scaffolds was mainly determined by their physical characteristics, including pore size and surface area, and chemical characteristics (types and amounts of released ions during degradation). Considering porosities, the BV/TV of G‐40‐2 was ≈2.8 times higher than that of G‐80‐2 on day 90 after implantation. For scaffolds with different unit sizes, G‐60‐1.5 had a higher BV/TV and Tb. N, and a lower Tb. Sp, indicating the better osteogenic ability compared with other groups. G‐40‐2 and G‐60‐1.5 showed the best osteogenesis in scaffolds with different porosities and unit sizes respectively due to their suitable pore size (400 – 600 µm) and large surface area. Due to the limited amount of released ions during degradation, the influence of chemical characteristics on the in vivo osteogenesis of Zn‐2Mg scaffolds was not significant compared with physical characteristics.

## 5 Experimental Section

### Sample Preparation

**Figure 8:**
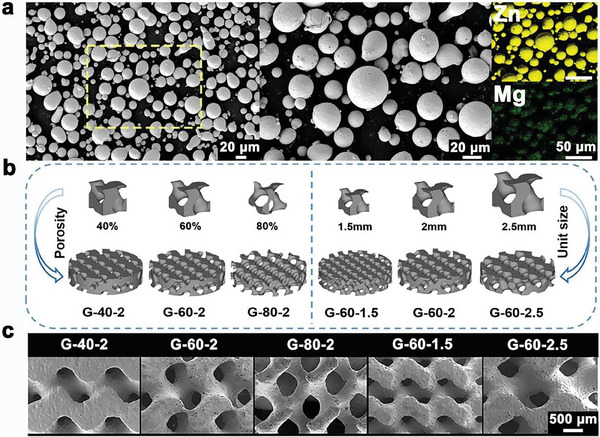
Fabrication of Zn‐2Mg scaffolds: a) morphology and element distribution of Zn‐2Mg powders by SEM observation and EDS analysis, b) the structure design of Zn‐2Mg scaffolds, and c) surface morphology of Zn‐2Mg scaffolds observed by SEM.

Zn‐2Mg alloy powders (Hebei Baotor New Material Technology Co., China) with a mean diameter (D50) of 25.55 µm were utilized for the fabrication of scaffolds. As shown in **Figure** **8**a, powders showed spherical morphology and uniform element distribution characterized by a scanning electron microscope (SEM, Zeiss, Germany) equipped with an energy dispersive spectrometer (EDS, Ametek, USA). An inductively coupled plasma optical emission spectrometer (ICP‐OES, iCAP6300, USA) was used for the analysis of chemical composition. The measured content of Mg in powders was 2.15 ± 0.01 wt.%.

The triply periodic minimal surface (TPMS) method was utilized to design scaffolds, which had zero mean curvature. Gyroid unit cells were generated with different dimensional sizes of 1.5, 2, and 2.5 mm and varying porosities of 40%, 60%, and 80%, as shown in Figure 8b. Scaffolds with a unit size of 2 mm and porosities of 40%, 60%, and 80% were denoted as G‐40‐2, G‐60‐2, and G‐80‐2. Scaffolds with a porosity of 60% and unit sizes of 1.5, 2, and 2.5 mm were denoted as G‐60‐1.5, G‐60‐2, and G‐60‐2.5. The fabricated scaffolds had different outline sizes, φ 6 × 6 mm^3^ for mechanical and in vitro degradation tests, φ 10 × 2.5 mm^3^ for in vitro biocompatibility and osteogenic ability evaluation, and φ 2.8 × 4 mm^3^ for in vivo experiments. Table 1 showed the structural characteristics of designed scaffolds with the dimension of φ 6 × 6 mm^3^. The pore size of the unit cell was defined as the diameter of the inscribed circle between struts of the unit cell in planer view.^[^ ^33^ ^]^ Strut diameter was defined as the minimum thickness of the periodic surface in the gyroid unit cell (Figure S5, Supporting Information).

Scaffolds were fabricated using a commercial L‐PBF system (BLT S210, China). The machine was equipped with a single‐mode ytterbium fiber laser (IPG YLR‐500, Germany) with a 70 µm focus spot diameter at the wavelength of 1070 nm. The oxygen content during the manufacturing process was kept below 120 ppm in an argon‐shielded chamber. Crucial L‐PBF process parameters included laser power (*P*), scanning speed (V_S_), hatching space (*H_S_*), and layer thickness (*D_S_*). Based on preliminary experiment results, the process parameters were set as *P* = 50 W, V_S_ = 500 mm ^−1^s, *H_S_* = 70 µm, and *D_S_* = 20 µm. After fabrication, scaffolds were polished in a solution containing 5% hydrochloric acid (Tongguang, China), 5% nitric acid (Hushi, China), and 90% ethanol (Fuchen, China) for 2 minutes. The specimens were then ultrasonically cleaned in 75% ethanol (Zhenyu, China) to remove residual acid and then air‐dried in an oven at 37 °C. The surface morphology of scaffolds was observed using SEM. As shown in Figure 8c, the surface of the polished scaffolds was smooth without unmelted powder adherence.

Mechanical and in vitro degradation tests: Compression tests were carried out using a universal testing machine (Shimadzu, AG‐X100KN, Japan) at a constant speed of 3 mm min^−1^ at room temperature. The compression direction was set to be parallel to the building direction. Three replicate samples were tested for the average value and standard deviation. In vitro immersion tests were performed in Hank's solution (Table S1, Supporting Information) at 37 °C for 90 days. Hank's solution was renewed every 3 days and pH values were recorded using a pH meter (Mettler FiveEasy, FE20, Switzerland). To obtain the weight loss of scaffolds, samples were cleaned ultrasonically using 10% chromic acid (Aladdin, China), dried at 37°C, and subsequently weighed. SEM and EDS were adopted for the analysis of degradation products. The samples after the immersion of 7, 14, 30, 60, and 90 days were compressed to obtain the change in mechanical behavior of scaffolds after degradation.

### In Vitro Biocompatibility

*Cell viability tests*: Cell viability evaluation was conducted according to ISO 10993–5. Mouse osteoblast precursor cells (MC3T3‐E1) were chosen to investigate the in vitro cell responses. All samples were sterilized via Co‐60 irradiation, immersed in an equivalent volume of culture medium, and then incubated at 37 °C for 24 ± 0.5 h in a cell incubator. Subsequently, the supernatant was collected to prepare a 100% concentration extract. The ion concentration in the extract was measured by ICP‐OES. Considering the slow in vivo degradation rate of Zn‐based BMs and the rapid metabolic exchange of corrosion products, subsequent in vitro experiments utilized 1‐fold and 2‐fold diluted extract.^[^ ^34^ ^]^ The Cell Counting Kit‐8 (CCK‐8, APExBIO, USA) was employed to assess the viability of MC3T3‐E1 cells. MC3T3‐E1 cells were seeded into the 96‐well plate. The medium was then replaced by the extracts for incubation over 1, 3, and 5 days. At these specific time points, a serum‐free medium was used to replace the original medium. Then 10 µL of CCK‐8 solution was added to the serum‐free medium. The medium containing the CCK‐8 solution was incubated at 37 °C in a dark environment for 10 – 30 minutes. The optical density (OD) value was recorded using a microplate reader (Thermo Fisher Scientific, USA) at 450 nm. Then, cell viability was calculated according to the equation below: Cell viability = [(OD value of experimental wells – OD value of blank wells)/(OD value of control wells – OD value of blank wells)] × 100%.

For the live/dead staining, MC3T3‐E1 cells were incubated in a 24‐well plate and co‐cultured with the 1‐fold and 2‐fold diluted extracts. The cells were subsequently stained with a Live/Dead cell staining kit (Beyotime, China), and the live (green) and dead (red) cells were observed by fluorescence microscope to assess cellular survival and morphology.

In vitro osteogenic differentiation: Cells were seeded in a 12‐well plate at a density of 3 × 10^4^ mL^−1^. When cells attach to the bottom of the plate, the culture medium was discarded and replaced with osteogenic induction extracts. The osteogenic induction extracts were composed of 10 mM β‐glycerophosphate (Sigma, USA), 50 µg mL^−1^ vitamin C (Solarbio, China), and 0.1 mM dexamethasone (Solarbio, China). After a 14‐day incubation, cells were gently rinsed with PBS. The cells were lysed using inhibitor‐free Western and IP cell lysates (Beyotime, China), and total protein was subsequently extracted. The total protein content was determined using a bicinchoninic acid protein assay kit (BCA, Thermo Fisher, USA), and the alkaline phosphatase (ALP) activity of MC3T3‐E1 was detected with an ALP detection kit (Beyotime, China). ALP staining was also performed by utilizing the ALP kit (Beyotime, China) and imaged by a high‐quality microscope. On day 21 of co‐culture, alizarin red staining (Solarbio, China) was performed to evaluate the formation of calcified nodules. The stained images were obtained by a high‐quality microscope. Finally, calcified nodules were dissolved by adding 10% cetylpyridinium chloride extract (Solarbio, China), and the OD value was measured at 562 nm.

### In Vivo Tests

*Surgical procedure*: All animal operations were approved by the Animal Ethics Committee of the Beijing Keyu Animal Breeding Center (KY20220120006). 45 male healthy SD rats (200 – 250 g) were anesthetized with an intraperitoneal injection of 2% xylazine (10 mg kg^−1^, Fluorochem, UK) and ketamine (10 mg kg^−1^, Dr. Ehrenstorfer GmbH, Germany). A hole (φ 3 mm) was drilled to establish a bone defect on the femoral condyle, then scaffolds were implanted into the defects. The rats were euthanized after 30, 60, and 90 days of implantation to collect femurs.

### Micro‐Computed Tomography (Micro‐CT) Analysis

At 30, 60, and 90 days after implantation, intact femurs of rats were collected and fixed in 4% paraformaldehyde. The femur specimens were scanned using a Micro‐CT scanner (Zeiss Xradia 520 Versa, Germany, 120 kV, 66.7 µA). Subsequently, a three‐dimensional (3D) image was reconstructed using CTvox 3.0 software (Bruker, Germany). The 0.4‐mm area around the implant was set as the region of interest (ROI), and histomorphometric indexes, including trabecular separation (Tb.Sp), trabecular number (Tb.N), bone volume fraction (BV/TV), and trabecular thickness (Tb.Th), were calculated. Finally, the designed sample volume was used as a baseline, and the in vivo sample volume change after degradation was calculated based on a Micro‐CT 3D image.

### Histological Evaluation

The fixed rat femur was dehydrated and embedded in methyl methacrylate resin. Each specimen was sectioned along the femur's horizontal plane to obtain 3 – 4 slices with a thickness of 200 µm. These slices were then further ground to 100 µm thickness, stained with methylene blue‐basic fuchsin, and imaged using a high‐resolution microscope. Image J image analysis software was utilized to semi‐quantitatively analyze the area of new bone tissue in hard‐tissue slice images. In terms of biosafety evaluation, the hearts, livers, spleens, lungs, and kidneys of the rats were embedded in paraffin, and cut into 5‐µm‐thick sections for H&E staining for potential pathological change observation.

### Statistical Analysis

All quantitative data were represented as mean ± standard deviation (SD). The statistical significance of the data was evaluated by one‐way analysis of variance (ANOVA) using SPSS 23.0 statistical software (IBM, USA). *p < 0.05 were considered statistically significant.

## Conflict of Interest

The authors declare no conflict of interest.

## Author Contributions

X.W., A.L., Z.Z. contributed equally to this work and shared the first authorship. X.W., A.L., Z.Z., performed conceptualization, investigation, data curation, wrote the original draft. D.H. performed fabrication of scaffolds. Y.L. performed investigation, data curation. J.D. performed structure design. X.J. perfomed data curation. H.D. performed investigation. Y.Z. performed supervision, resources. P.W., Y.L. performed supervision, resources, wrote – review & edited the original draft.

## Supporting information

Supporting Information

## Data Availability

The data that support the findings of this study are available from the corresponding author upon reasonable request.
