# Supplementary material for: Additively Manufactured Zn‐2Mg Alloy Porous Scaffolds with Customizable Biodegradable Performance and Enhanced Osteogenic Ability
Source: Adv Sci (Weinh). 2023 Dec 7;11(5):2307329. doi: 10.1002/advs.202307329 (PMC10837348; doi:10.1002/advs.202307329)
Supplement: Supplementary file 1 — Supporting Information [file ADVS-11-2307329-s001.pdf]

ADVANCED SCIENCE
Open Access

Supporting Information

for *Adv. Sci.*, DOI 10.1002/advs.202307329

Additively Manufactured Zn-2Mg Alloy Porous Scaffolds with Customizable Biodegradable Performance and Enhanced Osteogenic Ability

Xuan Wang, Aobo Liu, Zhenbao Zhang, Dazhong Hao, Yijie Liang, Jiabao Dai, Xiang Jin, Huanze Deng, Yantao Zhao, Peng Wen* and Yanfeng Li**

Supplementary materials

Additively manufactured Zn-2Mg alloy porous scaffolds with customizable biodegradable performance and enhanced osteogenic ability

Xuan Wang[a,b,1], Aobo Liu[c,d,1], Zhenbao Zhang[b,1], Dazhong Hao[c,d], Yijie Liang[b], Jiabao Dai[c,d], Xiang Jin[b], Huanze Deng[b], Yantao Zhao[b,e,f***], Peng Wen[c,d**], Yanfeng Li[b, a*]

[a] Postgraduate Training Base, Jinzhou Medical University and The Fourth Medical Centre, Chinese PLA General Hospital, Beijing, 100048, China.

[b] Department of Stomatology, the Fourth Medical Centre, Chinese PLA General Hospital, Beijing, 100048, China.

[c] State Key Laboratory of Tribology in Advanced Equipment, Beijing, 100084, China.

[d] Department of Mechanical Engineering, Tsinghua University, Beijing, 100084, China.

[e] Senior Department of Orthopedics, the Fourth Medical Center, PLA General Hospital, Beijing, 100048, China.

[f] Beijing Engineering Research Center of Orthopedics Implants, Beijing, 100048, China.

[1] Equal contribution, Joint first authors: Xuan Wang, Aobo Liu, and Zhenbao Zhang.

Corresponding authors: Yanfeng Li[*], Peng Wen[**], and Yantao Zhao[***].

1. Figures

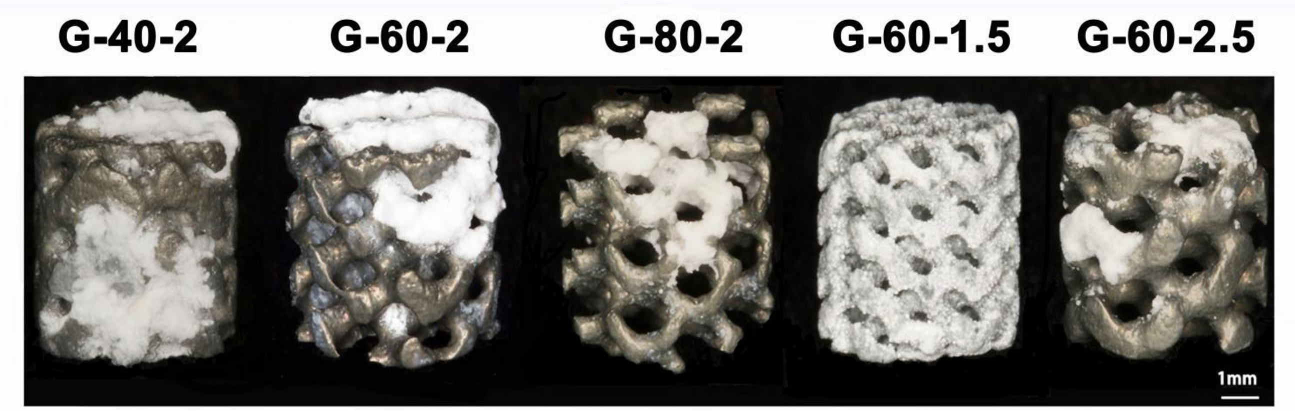

Figure S1. Appearance of Zn-2Mg scaffold during 90-day *in vitro* degradation experiment in Hank's solution.

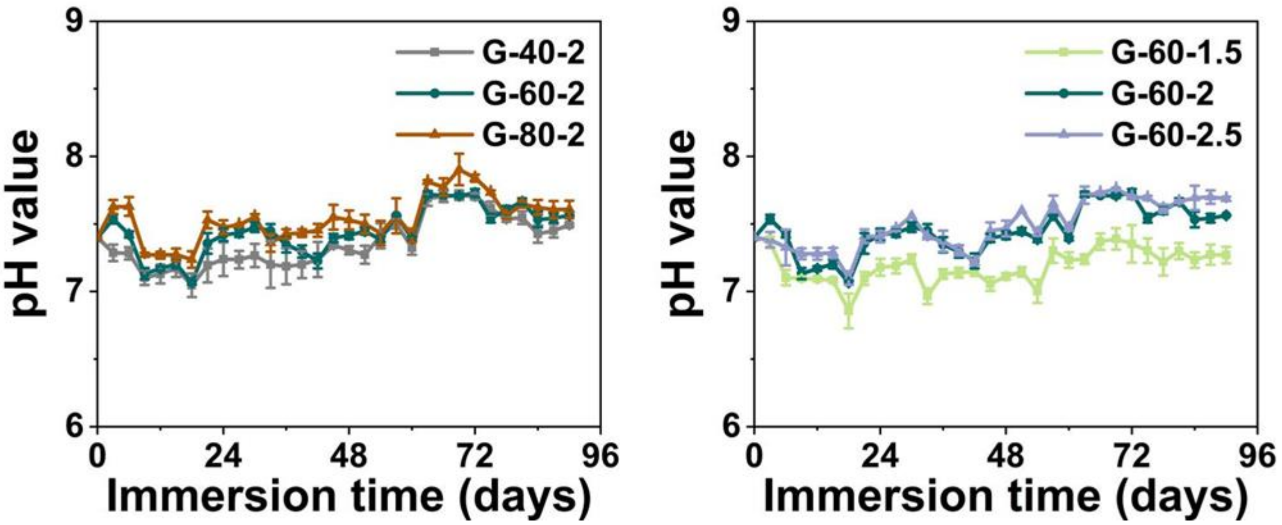

Figure S2. Changes in pH values during *in vitro* immersion tests.

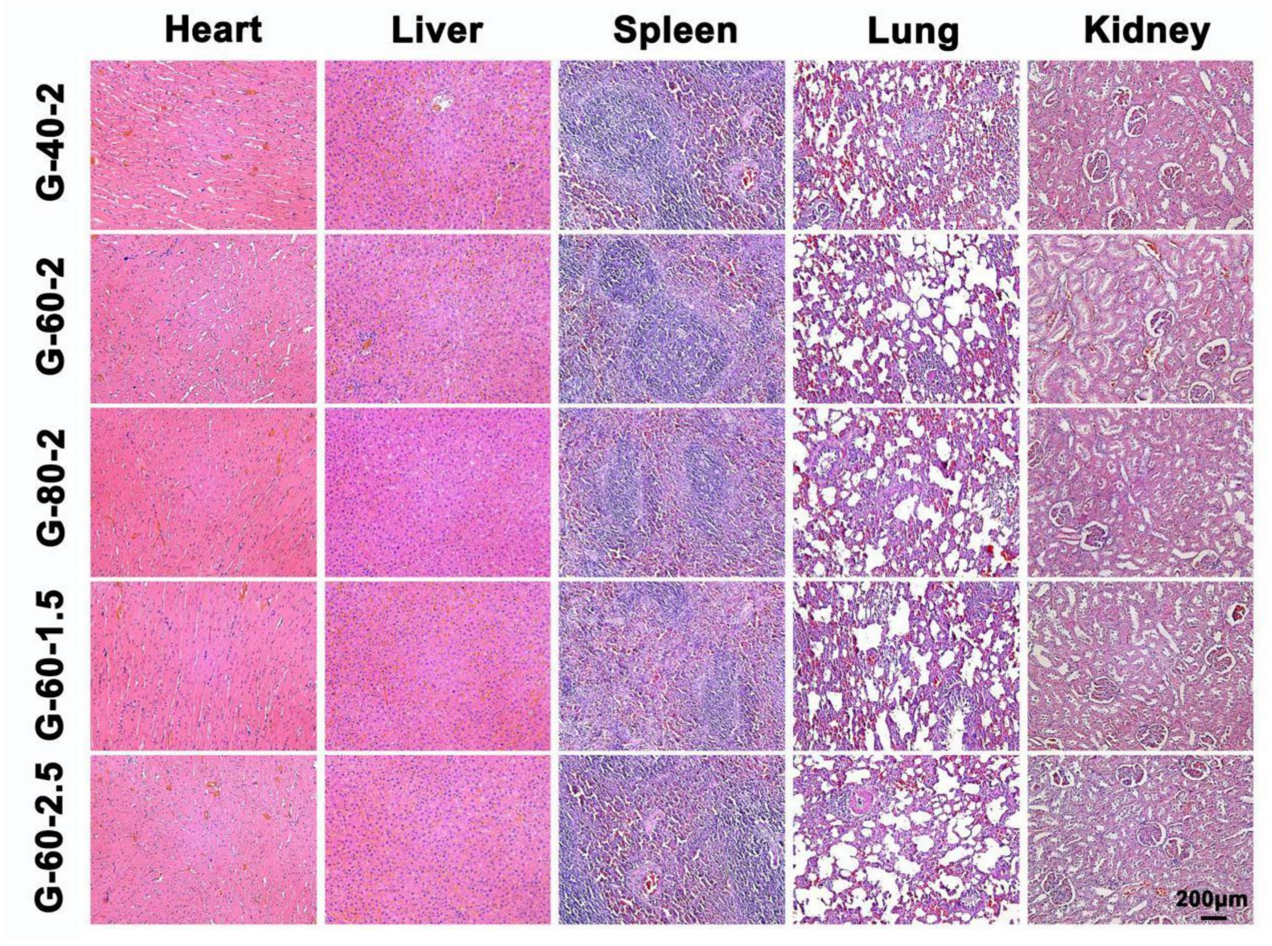

Figure S3. Histological evaluations of vital organs including heart, liver, spleen, lung, and kidney.

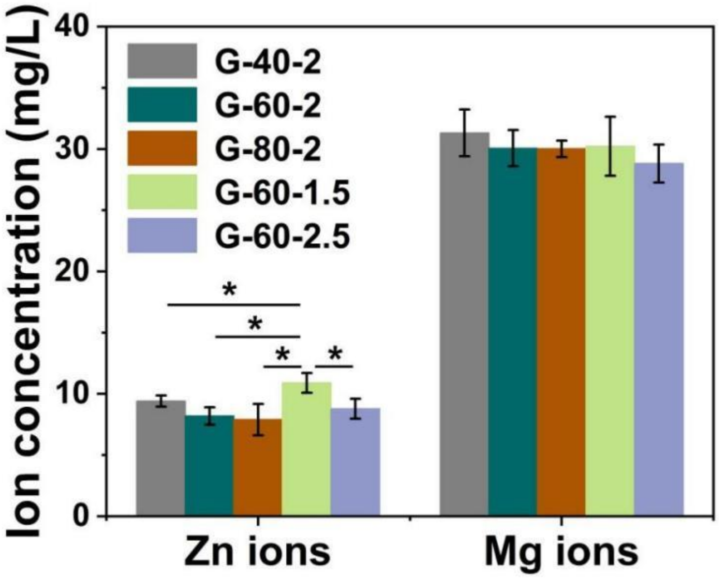

Figure S4. Ion concentration of the extracts.

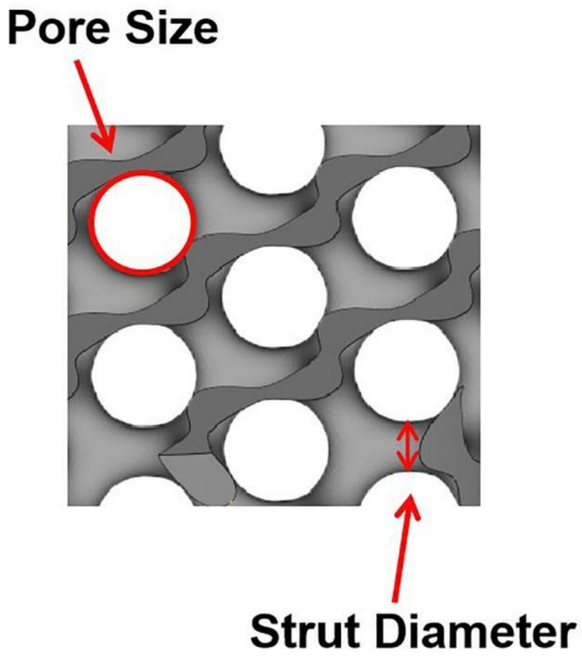

Figure S5. The definition of pore size and strut diameter.

2. Tables

Table S1. Composition of Hank's solution.

Composition	Na^+	K^+	Ca^{2+}	Mg^{2+}	Cl^-	HCO_3^-	HPO_4^{2-}	SO_4^{2-}
Concentration (mM)	141.73	5.8	1.26	0.812	144.7	4.166	0.336	0.812
